# Furunculosis of the External Auditory Meatus

**Published:** 1907-07-13

**Authors:** 


### Otology.

### FURUNCULOSIS OF THE EXTERNAL AUDITORY MEATUS.

Sometimes the pain of a boil in the ear is comparatively slight, amounting merely to a little local tenderness. Very often it is severe enough to disturb sleep, and occasionally it is so acute that even a robust man soon becomes thoroughly ill.

### Pathology.

The essential cause is the staphylococcus pyogenes aureus, or albus in the hair follicles of the meatus. Follicular inflammation often begins without any obvious cause, in apparently healthy persons. Sometimes it is part of a general furuncular state, and sometimes may be observed in typhoid fever, diabetes, and other debilitating diseases. Local irritation is not infrequent; indeed a boil often complicates chronic otorrhœa, due to otitis media. Sometimes the cause is found in injuries to the ear inflicted by patients themselves when troubled with eczema or subjective pruritus. Probably men are more often affected than women. The condition is not common in children.

The process originates in the outer half of the meatus. A group of glands is inflamed, and forms a tender swelling. The focus enlarges and the central area sloughs; the slough is surrounded by pus, which in time points, and, if permitted, eventually bursts on the surface. As the slough disintegrates and is discharged with the pus the inflammation gradually subsides, and, in process of time, all swelling disappears.

### Symptoms.

The symptoms vary somewhat, according as the boil begins superficially or deeply. In the latter case the first symptom is increasing throbbing pain radiating to the head or neck. The pinna becomes tender when touched, and the pain may be almost maddening if the meatus is moved. With movements of the jaw the pain increases so that the patient may be almost afraid to eat. Sleep is interfered with, and the patient soon comes to wear an expression indicative of pain and fatigue. To examine the ear a good light should be obtained, and reflected into the meatus. At first very little may be seen to account for the great pain, especially when the inflammation is deep seated in origin. Sooner or later the side of the meatus affected becomes flatter, and the canal narrower. In time the swelling increases until an elevation projects into the meatus, and the lumen is almost or quite closed. Provided there be no meatal débris, it may be easy to view the membrane and parts of the canal deep to the swelling, in the early stages. This examination should be made for the purpose of excluding swellings of the meatus due to mastoiditis.

The most frequent situation for a boil is the posterior or inferior wall or the inner surface of the tragus. Although the lymphatic glands of the neck are seldom involved, those over the parotid are frequently enlarged and tender, and it is not very uncommon to find the glands on the mastoid process affected too. It is most important to recognise that the post-auricular furrow is often swollen and œdematous, especially in boils on the posterior meatal wall. This must not be mistaken for superficial mastoiditis, a distinction not always easily made. Furuncles often recur in the meatus one after the other. Though generally single, several at a time are occasionally met with.

### Treatment.

The first indication is the relief of pain. This is often very severe in character and robs the patient of rest. Opiates and other soporifics are, however, to be avoided if possible. Free incision into the most tender area will give certain and speedy relief. The most tender spot must be determined by carefully touching with a probe. Even when there is obvious swelling the rule should be to incise through the point of maximum tenderness. The incision must be made sufficiently deep, and must be made right down into the inflamed area. Local anæsthetics in the case of deep-seated furuncles are generally disappointing. Nitrous oxide gas should be given in these cases. In addition to local treatment, it is always helpful to administer an efficient purgative at the outset, preferably a mercurial one followed at an interval of four to six hours by a saline draught. This, in addition to its immediate effect on the inflammatory processes, is valuable in preventing recurrence of the trouble.

When the furuncle is of the superficial variety, and the pain less severe, we may use a local application. One of the most satisfactory and certain in action is glycerine of carbolic acid (acidum carbolicum liquefactum added to glycerine, 1:4) applied on a pledget of wool until the pain is relieved. The pledget should then be removed and a hot fomentation placed over the side of the head. In the later stages, when the furuncular abscess is about to burst, the incision should be made by transfixion with a strong, sharp-pointed knife. Even when the abscess has burst an incision will be indicated to hasten the cure, by giving free exit to the slough. If this is slow to separate, and there is a tendency to sprouting granulations with persistent infiltration lasting some weeks, the curette can be made very effective use of.

In mild cases boric acid in alcohol, 1:20, used in the form of drops two or three times a day, will often cause a small boil to disappear in a few days.

### Opsonins and Vaccines.

If in spite of attention to the general health, change of air, and so forth furuncles still recur, one must consider the anti-staphylococcic vaccine treatment. We have known more than one case in which this alone led to a permanent cure.

